# Pharmacogenomics Education and Knowledge Assessment in Healthcare Curricula: A Scoping Review of the Middle East and North Africa Region

**DOI:** 10.1002/prp2.70121

**Published:** 2025-07-11

**Authors:** Asmaa Ibrahim, Reem Alenany, Loulia Bader, Fatima Nazar, Sara Abulola, Ola Almasalmeh, Sadaf Riaz, Hilyatuz Zahroh, Fatima Mraiche

**Affiliations:** ^1^ College of Pharmacy Qatar University Doha Qatar; ^2^ Department of Pharmacology, Faculty of Medicine & Dentistry, College of Health Sciences University of Alberta Edmonton Alberta Canada

**Keywords:** healthcare professionals, Middle East, North Africa, pharmacogenomics education, pharmacogenomics knowledge

## Abstract

Pharmacogenomics (PGx) is a crucial part of precision medicine; however, its integration into clinical practice has been slow, primarily due to knowledge gaps regarding pharmacogenomics among healthcare professionals (HCPs). Pharmacogenomics education is considered the most influential barrier to the successful implementation of PGx. In the Middle East and North Africa (MENA) region, several studies have highlighted the lack of PGx education among students and HCPs. This scoping review aims to provide an overview of the existing literature on how PGx is delivered in the curricula and its impact on knowledge acquisition among students and HCPs in the MENA region. A database search of PubMed, Embase, and CINAHL was conducted up to June 2023. Outcomes included PGx education availability, curriculum placement, mode of delivery, and knowledge level. Seven cross‐sectional studies were identified (2014–2023), with 57% from the Gulf region. PGx education is mostly taught to undergraduates (71%) through didactic lectures (100%), with only a few studies reporting a standalone course (29%). Participants demonstrated low (43%) to moderate (43%) knowledge levels, assessed using objective scales or subjective self‐assessment after completion of the course. Reported barriers to implementing PGx education included knowledge gaps, economic constraints, and system sustainability. PGx education in the MENA region is delivered mainly as an integrated course in professional programs including pharmacy and medicine, and the overall knowledge level was low to moderate. Future research could focus on providing more detailed reporting of the PGx educational landscape, developing standardized assessment tools, and evaluating actual knowledge acquisition among different professions.

## Graphical abstract

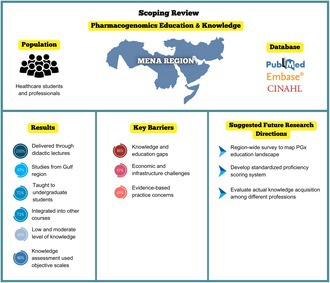

**Abbreviations**
CPD: continuing professional development
ELSI: ethical, legal, and social issues
HCP: healthcare professional
ISP: International Society for Pharmacogenomics
MENA: Middle East and North Africa
PGx: pharmacogenomics

## 1 Introduction

Pharmacogenomics (PGx) is a key component of precision medicine, which aims to tailor prevention, diagnostics, and therapeutics based on individuals' genetic factors [1]. The growing clinical relevance of PGx is reflected in the increasing number of drugs with PGx information on their labels [2, 3, 4]. Additionally, over 70 healthcare systems worldwide have been implementing genetic tests to guide clinical prescribing [2, 5, 6]. This implementation is of particular importance in diverse populations, such as those in the Middle East and North Africa (MENA) region, where genetic variability can influence drug responses. The rapid integration of PGx into clinical settings necessitates competent healthcare professionals (HCPs) who can interpret and apply PGx data to clinical decisions [7].

Implementing PGx into clinical practice faces several barriers. PGx education is considered the most influential barrier as it influences all those involved in personalized medicine implementation. The lack of PGx knowledge is secondary to insufficient training and educational programs for HCPs and other stakeholders. To address this issue, it has been recommended that PGx education be incorporated into the curricula of HCPs and that training be provided to them to increase their knowledge and confidence in delivering PGx care to patients [8, 9]. The curricula and training should ensure that graduates possess the knowledge and skills for coordinating and collaborating with an interdisciplinary team of HCPs in evaluating genetic information [10].

In 2005, the International Society for Pharmacogenomics (ISP) recommended integrating PGx teaching into the core pharmacology curriculum of medical, pharmaceutical, and healthcare schools [11]. They further recommended implementing PGx as a standalone elective or, ideally, as a mandatory subject in these schools. The recommendations also include the provision of at least 4 h of PGx teaching per program, with an ideal extension to 8 h, in addition to more extensive incorporation in pharmaceutical, life sciences, and public health schools. They also suggested using different educational tools such as review articles, web resources, and case studies and creating open‐access, comprehensive, web‐based tutorials, including online lectures, presentations, and manuscripts [11].

Several pharmacy programs in the MENA region, such as Qatar University [12], Beirut Arab Univer [13], and Jordan University of Science and Technology [14], are accredited by international bodies including the Accreditation Council for Pharmacy Education (ACPE) and the Canadian Council for Accreditation of Pharmacy Programs (CCAPP). Notably, both organizations require PGx as part of their standards, making it a key component of pharmacy education. The ACPE 2025 accreditation standards list PGx as a required subject, both as a standalone topic under pharmaceutical sciences and integrated into pharmacotherapy within clinical sciences [15]. Similarly, CCAPP Standard 4 ensures PGx is covered alongside other core pharmaceutical sciences [16].

According to a global survey conducted in 2019 (universities from 7 continents including Asia and Africa), most healthcare programs (87%) including medicine, pharmacy, nursing, and other health professions have integrated PGx as part of their pharmacology curriculum. The majority (63%) of the programs offer at least the recommended minimum teaching hours (i.e., 3–4 h). Specifically, 75.9% of pharmacy programs and 80% of graduate programs (PhD, MSc) meet these recommendations, whereas only 48.5% of medical programs do. Furthermore, the most commonly used educational tools for teaching PGx are original research papers (70.8%), followed by internet databases (48.6%) and textbooks (41.7%) [17].

In the MENA region, published literature regarding PGx education is scarce. A study from the American University of Beirut (AUB) mentioned that in the second year of the undergraduate pharmacy program, PGx is implemented as part of the pharmacology and toxicology course with two 1‐h didactic lectures devoted to drug metabolism and PGx. This is accompanied by a 90‐ to 120‐min exercise in which students apply the acquired knowledge in solving clinically relevant scenarios. In the fourth‐year, PGx principles are reinforced in several sessions of the clinical pharmacology course [18]. However, the impact of these curricula on students' knowledge was not assessed. Furthermore, Qatar University offers a standalone elective PGx course in the fourth‐year of the undergraduate pharmacy program, with a mix of didactic lectures, lab‐based cases, and appropriate utilization of PGx resources. Given the few studies investigating PGx education in the MENA region, a comprehensive scoping review of PGx education across this region and its impact on knowledge is currently lacking. For stakeholders to put PGx into practice in the MENA region, it is imperative to understand the current state of PGx education and its incorporation into HCP curricula. A preliminary search of the literature revealed a limited number of publications addressing how PGx education is delivered and the knowledge of HCPs about PGx in the MENA region.

This scoping review was conducted to summarize the existing literature about the delivery of PGx education in the MENA region (regarding availability, curriculum placement, mode of delivery, approach, and barriers) and its impact on knowledge levels among students and HCPs. Additionally, we aim to identify key barriers to pharmacogenomics implementation in clinical practice and recommendations to improve pharmacogenomics education in the MENA region.

## 2 Methods

### 2.1 Study Design

This scoping review is described according to the Preferred Reporting Items for Systematic Reviews and Meta‐Analyses Extension for Scoping Reviews (PRISMA‐ScR) available from: https://www.prisma‐statement.org/scoping.

### 2.2 Inclusion and Exclusion Criteria

This review included peer‐reviewed studies that used experimental study designs such as randomized and non‐randomized controlled trials, and observational study designs such as cohort, case–control, and cross‐sectional studies. Secondary studies including narrative, scoping, and systematic reviews, as well as gray literature (e.g., white papers and conference abstracts) were excluded. Participants included undergraduate, postgraduate (master's and doctoral degrees), and alumni students enrolled in clinical (pharmacy, medicine, dentistry, nursing) or non‐clinical (public health, biology, biomedical sciences, physiotherapy) programs, as well as practicing HCPs.

Delivery of PGx knowledge within the curricula included but was not limited to availability, mode of delivery (lab, cases, course), approach (integrated, standalone, mandatory, elective), circular placement, and year of delivery.

The inclusion criteria were as follows: (1) studies describing educational interventions related to the delivery of PGx knowledge within the curricula of clinical and non‐clinical programs; (2) studies published in English from database inception up to June 2023; (3) studies conducted within the MENA region; (4) and studies evaluating both PGx knowledge (exposure to PGx content in the curriculum, knowledge score, or level) and (5) delivery aspects (e.g., availability, mode of delivery, or approach).

The exclusion criteria included secondary literature (narrative reviews, scoping reviews, systematic reviews) and gray literature (white papers, conference abstracts); and educational interventions delivered through continuing professional development (CPD) courses.

### 2.3 Search Strategy

PubMed, Embase, and CINAHL databases were selected based on the preliminary search and subjected to a systematic search for relevant literature. Google scholar was only used for the preliminary search.

The search strategy was defined through the principles of a systematic search using the PCC (population [MENA region], concept [PGx education], context [delivery and knowledge]) framework. The search strategy employed for this scoping review uses a combination of specific terms and database‐specific search operators to comprehensively identify relevant literature. The detailed search terms are presented in Table 1.

**TABLE 1:** Search strategy for each database searched.

PubMed	(middle east [MeSH Terms] OR africa, northern [MeSH Terms]) AND (pharmacogen* OR precision medicine OR medicine, personalized [MeSH Terms]) AND (education OR curricul* OR teaching OR delivery OR learning)
Embase	(‘Middle East’/exp. OR ‘North Africa’/exp) AND (‘pharmacogenetics’/exp. OR ‘pharmacogenomics’/exp. OR ‘personalized medicine’/exp) AND (‘education’/exp. OR ‘curriculum’/exp. OR ‘teaching’/exp. OR ‘learning’/exp)
CINAHL	MW (Algeria or Bahrain or Egypt or Iran or Iraq or Palestine or Jordan or Kuwait or Lebanon or Libya or Morocco or Oman or Qatar or Saudi Arabia or Syria or Tunisia or United Arab Emirates or Yemen or Sudan or Djibouti or Turkey) AND TX (pharmacogenetics or pharmacogenomics or precision medicine or personalized medicine) AND TX (curriculum or curricula or teaching or learning or education)

### 2.4 Screening Process

The retrieved records were exported to EndNote 20 software (Clarivate) for duplicate removal and then to the Rayyan.ai platform [19] for title/abstract and full‐text screening. Each record was screened by two reviewers, and conflicts were resolved by discussion or by consulting a third reviewer.

### 2.5 Data Collection Process (Extraction)

Relevant data were extracted from the included studies by two independent reviewers using Microsoft Excel. The data extracted included the characteristics of the study and the participants (country, year, objective, study design), sample size, and profession. Study outcomes including availability, mode of delivery, approach, and knowledge level were also extracted.

## 3 Results

### 3.1 Selection of Studies

**FIGURE 1:**
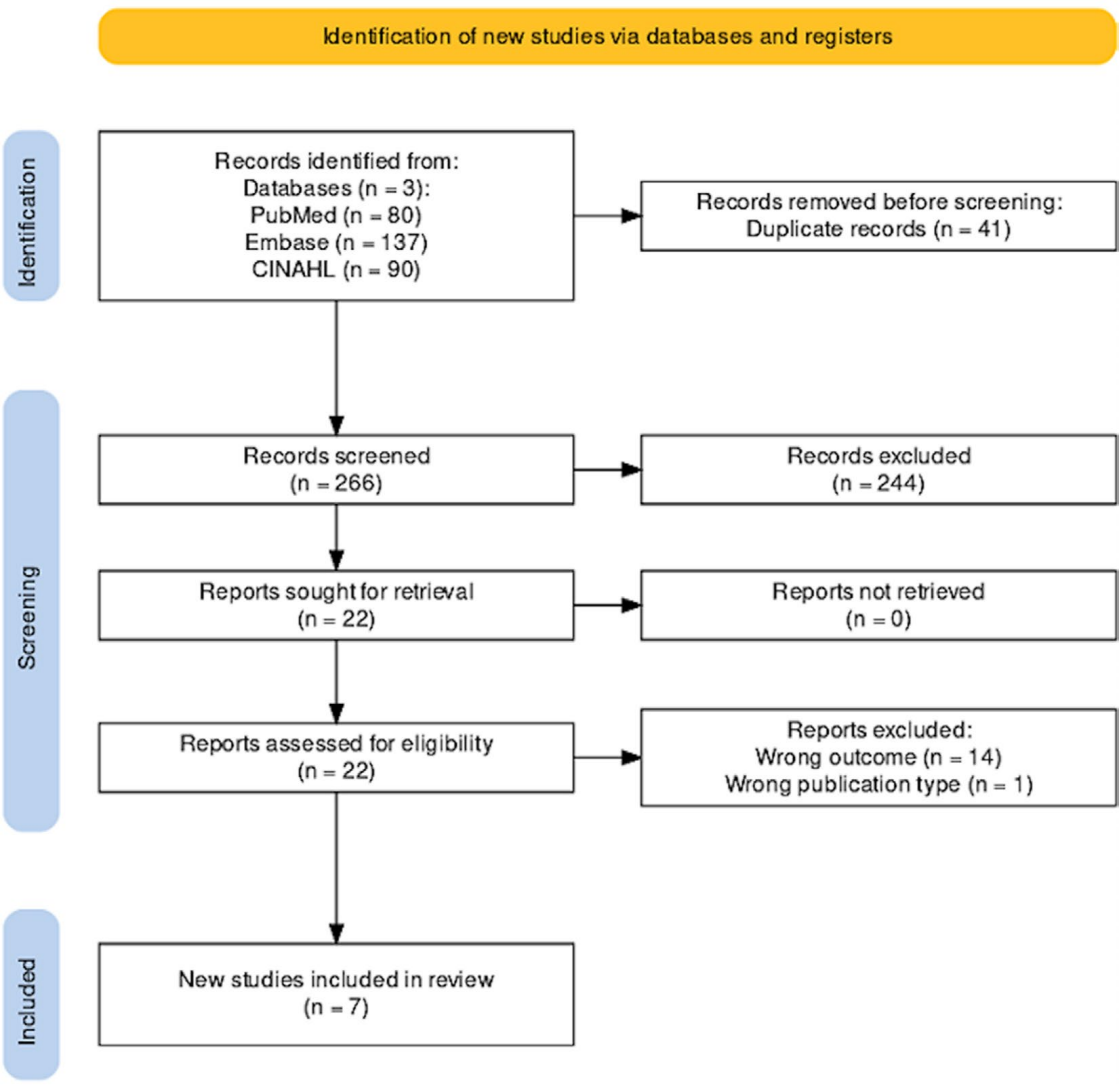
Preferred Reporting Items for Systematic Reviews and Meta‐Analyses (PRISMA) flowchart.

A summary of the studies included is presented in Table 2. A total of 307 records were identified through the search in PubMed (*n* = 80), Embase (*n* = 137), and CINAHL (*n* = 90), of which 41 were duplicates. After screening the titles and abstracts of 266 articles, 244 of them were deemed irrelevant. The remaining 22 articles were retrieved for full‐text assessment. After applying the exclusion criteria, 15 articles were excluded, leaving 7 articles to be included in the review. A PRISMA flow diagram of the selection process is presented in Figure 1.

**TABLE 2:** Summary of the included articles.

Study	Country	Study design	Profession	Curricular placement	Mode of delivery	Approach	Knowledge level
Al‐Eitan et al. (2014) [20]	Jordan	Cross‐sectional survey	Pharmacy, Medicine	Year 1/2/3/4/5 but mostly in the second year, Master of Science degree (MSc) and Doctor of Philosophy degree (PhD)	Didactic, lab, experiential rotation	Standalone or mixed, mostly < 10 h, mostly elective	Higher with PGx users
Albassam et al. (2018) [21]	Kuwait	Cross‐sectional survey	Pharmacy, Medicine	Undergraduate	Didactic	Integrated in biochemistry and pharmacology courses (over few hours), mandatory	Low
Rahma et al. (2020) [22]	United Arab Emirates	Cross‐sectional survey	Pharmacy, Medicine, Health Science, Public Health	Starting from second year	Didactic	Standalone genetic and PGx courses	Fair
Bagher et al. (2021) [23]	Kingdom of Saudia Arabia	Cross‐sectional survey	Pharmacy	Undergraduate and postgraduate	Didactic	Integrated in pharmacology courses, mandatory	Low
Yehya et al. (2021) [24]	Jordan	Cross‐sectional survey	Pharmacy, Medicine	Within the first 3 years	Didactic	Integrated as a chapter (4 lectures, 6 h) within introduction to pharmacology, mandatory	Moderate
Arafah et al. (2022) [25]	Kingdom of Saudia Arabia	Cross‐sectional survey	Pharmacy	Throughout the years	Didactic	Integrated in a comprehensive pharmacology program, mandatory	Fair
Jarrar et al. (2023) [26]	West bank of Palestine	Cross‐sectional survey	Pharmacy, Medicine, Nursing, Physiotherapy	Undergraduate and postgraduate	Didactic	Integrated in pharmacology and pharmacotherapy courses, mandatory	Low

### 3.2 Characteristics of Studies

The date of publication ranged between 2014 and 2023. Of the included articles, the majority (57%) were published between 2020 and 2022. Most articles originated from the Gulf region (57%), with the rest coming from the Levant region (43%), and none were from the North African region. All articles employed a cross‐sectional survey design and included a diverse range of professions. All the articles identified included the pharmacy profession, 71% included the medical profession, and 14% included health sciences, nursing, and physiotherapy (Table 2).

### 3.3 PGx Education Practices

The included studies described various approaches to integrating PGx education into HCPs curricula. The majority (71%) of the articles reported that PGx was integrated throughout the years of the undergraduate degree [20, 21, 23, 25, 26], whereas 29% reported offering PGx courses during the early years of the program [22, 24]. Only one article reported offering PGx in the second year [22]. Forty‐three percent of the articles reported PGx training at the postgraduate level. The most common mode of delivery was didactic lectures, which were reported in all articles. One article also employed laboratory sessions and experiential rotations in addition to the didactic lectures. PGx was either integrated into other courses, such as pharmacology (71%), or offered as standalone courses (29%).

### 3.4 PGx Knowledge Assessment

The included articles assessed the level of PGx knowledge of participant HCPs using cross‐sectional surveys (Table 3). Knowledge level was classified as ‘low’, ‘moderate/fair’, or ‘high’ depending on the participants score in the knowledge tests. Each study had different number and type of questions and cut‐off points for ‘low’, ‘moderate’, and ‘high’. In this review, we reported the levels reported by the authors and did not standardize the cut‐off points.

**TABLE 3:** Level of Knowledge reported and the scales used for assessment in the included articles.

Type of scale	
Subjective self‐assessment	[20]
Objective scale	
Knowledge on PGx applications	[21, 23]
Foundational knowledge of PGx	[21, 22, 23, 25, 26]
Foundational knowledge of genetics	[22, 24]
Ability to apply PGx to clinical case scenario	[24]
Reported level of knowledge in the articles	
High	—
Moderate	[22, 24, 25]
Low	[21, 23, 26]
Not reported	[20]

None of the articles reported a high level of PGx knowledge. Specifically, 43% reported moderate knowledge, and 43% reported a low level of knowledge. One article did not report the PGx knowledge level of the participants but described the self‐assessed knowledge in PGx users as being more sufficient than non‐users (*p* = 0.003) [20].

The assessment methods in the studies varied (Table 3). In one article (14%) [20], the authors used a subjective self‐assessment in which the participants rated their perceived level of knowledge on a 5‐point Likert scale, with 1 being very little knowledge and 5 being highly knowledgeable. Respondents rated their PGx knowledge as inadequate with a mean (±SD) score of 2.0 (±1.0). The remaining articles (86%) employed objective scales for knowledge assessment. Two articles [21, 23] assessed knowledge of participants on PGx applications using a 5‐item multiple‐choice questionnaire. A score of 1 was given for correct answers, and 0 for incorrect answers or ‘do not know’ where applicable. Most participants (> 50%) in both studies scored only one to two items correctly, indicating ‘low’ knowledge. Five articles [21, 22, 23, 25, 26] assessed foundational knowledge of PGx using multiple‐choice and true/false questions. The mean score of participants in articles [21, 23, 26] were 2.25/5, 2.4/5, and 5.7/12, respectively, indicating low knowledge, whereas articles [22, 25] reported moderate/fair knowledge with mean scores of 5.4/9 (±2.7) and 4.6/8 (±1.5), respectively. Only one article [24] assessed the ability to apply PGx information in answering a clinical case scenario using a 4‐item MCQ. The overall knowledge level was judged as moderate, as 77.3% of respondents answered 2–3 questions correctly. Articles [22, 24] had additional components to assess foundational knowledge of genetics. Sample statements from objective scales to assess PGx knowledge are provided in Table 4. Table 5 summarizes the areas of knowledge assessment in the included studies based on Soueid et al. classification. Soueid et al. used 4 areas to assess the impact of educational interventions on knowledge and confidence including foundational knowledge, data interpretation, patient communication, and HCP communication [27].

**TABLE 4:** Example statements on objective scales.

Type of PGx knowledge assessed	Example statements
Knowledge on PGx applications	“PGx has an important role in individualizing response to medications”
	“PGx has an important role in identifying drug–drug interactions”
Foundational knowledge of PGx	“The package insert for warfarin includes a warning about altered metabolism in individuals who have specific genetic variants”
	“Genetic determinants of drug response change over a person's lifetime”
	“Some patients have a high risk of drug toxicity due to inherited genetic variants”
Foundational knowledge of genetics	“Humans have 48 chromosomes”
	“Adenine (A) only pairs with Cytosine (C) and Thymine (T) only pairs with Guanine (G)”
Ability to apply PGx to clinical case scenario	Case study question: “Warfarin is mainly metabolized by the metabolizing enzyme: (A) CYP2C9; (B) CYP3A4; (C) CYP2D6; (D) CYP3A5; (E) I do not know”
	Follow‐up question: “Based on this result, the patient has: (A) Normal sensitivity, requires higher dose; (B) Decreased sensitivity, use the same dose; (C) Increased sensitivity, requires lower dose; (D) Decreased metabolism, requires higher dose; (E) I do not know”

**TABLE 5:** Areas of knowledge assessment reported in the included studies.

Studies	Areas of knowledge assessment			
	Foundational	Data interpretation	Patient communication	Healthcare professional communication
Al‐Eitan et al. (2014) [20]	No	No	No	No
Albassam et al. (2018) [21]	Yes, assessed the foundational knowledge of PGx and its applications	No	No	No
Rahma et al. (2020) [22]	Yes, assessed the foundational knowledge of PGx and genetics	No	No	No
Bagher et al. (2021) [23]	Yes, assessed the foundational knowledge of PGx and its applications	No	No	No
Yehya et al. (2021) [24]	Yes, assessed the foundational knowledge of PGx and its application	Yes	No	No
Arafah et al. (2022) [25]	Yes	No	No	No
Jarrar et al. (2023) [26]	Yes	No	No	No

### 3.5 Barriers to PGx Implementation

We used inductive content analysis to categorize the barriers into themes and sub‐themes (Table 6). The most frequently cited barriers (86%) were related to knowledge and education, including a lack of formal education and training at both undergraduate and postgraduate levels (three articles) and limited understanding of PGx knowledge (two articles). The remaining subthemes within knowledge and education barriers included limited accessibility of knowledge resources, lack of self‐confidence in practicing PGx, and low retention of previous knowledge.

**TABLE 6:** Barriers to PGx implementation identified in the articles.

Theme	Subtheme	References
Knowledge and Education Barriers	Lack of understanding of PGx knowledge	[23, 26]
	Lack of formal training and education, both at the undergraduate and postgraduate levels	[20, 21, 22]
	Accessibility of knowledge resources	[20]
	Lack of self‐confidence in practicing PGx	[23]
	Low retention of previous knowledge	[24]
Evidence‐based practice barriers	Absence of established PGx clinical guidelines	[21, 22]
	High‐quality evidence requirements	[20]
Economic and infrastructure barriers	High cost of testing	[20]
	Lack of testing services	[22]
	Complexity of PGx	[20, 24]
System sustainability challenges	Need for further education to promote personalized medicine	[20]

The second most common barrier was related to economics and infrastructure (57% of the articles). This topic included the complexity of PGx testing (29%), the high cost of testing (29%), and the lack of available testing services (29%).

Concerns about evidence‐based practice were identified in 43% of the included studies. This theme included the absence of established PGx clinical guidelines (29%) and the need for high‐quality evidence to implement PGx (14%). Finally, one article identified system sustainability challenges as a barrier, specifically the need for further education to promote personalized medicine.

### 3.6 Future Recommendations

Table 7 summarizes the future recommendations for improving PGx education and implementation. Using inductive content analysis, the data were categorized into themes and subthemes. The most common recommendation was to increase PGx education in HCPs curricula (86% of the articles) mainly by including PGx lectures, courses, and workshops at both the undergraduate and postgraduate levels (71%). Other recommendations included offering standalone PGx courses in the curricula (29%), integrating PGx applications into pharmacology courses (14%), and making PGx courses mandatory rather than elective (14%).

**TABLE 7:** Future recommendations identified in the articles for implementing PGx into practice.

Theme	Subtheme	Reference
Increase curricular education	Including PGx lectures, courses, and workshops into the syllabi of HCP students, at both the undergraduate and postgraduate level	[20, 21, 22, 25, 26]
	Offering standalone PGx courses in the curricula	[22, 25]
	Integrating PGx applications into pharmacology courses	[24]
	Mandatory incorporation of PGx courses into the core curriculum rather than elective	[20]
Collaborative governance for PGx education	Involving experts in the field and accreditation bodies for strategic curricular updates	[22]
Continuous professional development	Incorporate PGx topics into CPD programs	[21, 25]
	Integration of PGx training into practice through CPD	[21, 25]
Incorporation of PGx into clinical practice	Enhancing the translation of PGx knowledge into practice by fostering collaboration between academia and health settings	[22, 23]

Another key recommendation was continuous professional development (CPD), cited by 29% of the articles, with a focus on incorporating PGx topics into CPD programs and the integration of PGx training into practice through CPD. Similarly, 29% of the articles recommended improving the translation of PGx knowledge into practice by fostering collaboration between academia and health settings. One study recommended collaborative governance for PGx education, involving experts in the field and accreditation bodies for strategic curricular updates (Table 7).

## 4 Discussion

### 4.1 PGx Education in the MENA Region

This scoping review provides insights into the current state of PGx education in the MENA region, focusing on its delivery within the curricula of various programs and its impact on knowledge levels. The findings of this scoping review of seven articles showed that PGx education is primarily delivered within pharmacy and medical programs in the MENA region, often embedded within courses such as pharmacology. This aligns with the 2005 recommendations from the ISP to incorporate PGx into core pharmacology curricula of medical, pharmaceutical, and health professional programs [11].

Although the preferred year for delivering PGx education during undergraduate years was not specified, one study evaluated the impact of PGx delivery as a part of a new course, Biopharmaceutics and Pharmacogenomics, in the first‐year of pharmacy school. The study demonstrated significant improvement in students' PGx knowledge and their confidence in discussing PGx with other HCPs and patients (*p* = 0.001). Moreover, students demonstrated knowledge retention 6 months after course completion [28]. In addition, a study in the United Kingdom evaluated the inclusion of a required first‐year course, Principles in Genetics and Pharmacogenomics, and PGx integration into subsequent courses. First‐year pharmacy students reported feeling comfortable with their PGx knowledge and role in applying PGx. However, second‐ to fourth‐year students believed they should be able to apply PGx clinically after receiving additional integrated PGx courses [29]. These studies demonstrate that introducing PGx content early in the curricula may be beneficial to the application of the concepts and retention of the content.

A comparative study of PGx curricula among 75 U.S. colleges and schools of pharmacy reported that PGx was taught as part of integrated didactic lectures, with significant variations in hours delivered. Approximately 40.6% of programs covered 10 h, 42.0% covered 10–30 h, and 14.5% covered 31–60 h of PGx content [30]. In our review, two studies stated that the PGx content in programs is covered in less than 10 h [20, 24]. Furthermore, a global study of 248 schools (from 7 continents) of medicine, pharmacy, nursing, and health professions found that the majority of programs (87%) included PGx education as part of the pharmacology curriculum, with 63% offering at least the minimum ISP recommended number of PGx teaching hours [17] which is similar to our findings. Other studies also showed that PGx was most often integrated into pharmacology courses and rarely taught as a standalone subject [31, 32].

### 4.2 PGx Knowledge Levels

Our scoping review reveals a predominant trend of fair to low levels of PGx knowledge among students and HCPs. Students and HCPs often self‐assess their knowledge as inadequate or at low to moderate levels. Practice experience, coursework, and training in PGx significantly influence knowledge levels [22, 25]. Those with extensive practical experience, such as over 10 years of practice [21] or having completed coursework in PGx [25], tend to demonstrate better knowledge. Knowledge levels also vary based on the year of study, completion of training, and participation in internships or study abroad programs. Advanced‐level bachelor's students and those who completed PGx training reported higher knowledge levels. Additionally, those who completed an internship or study abroad program reported good or fair knowledge levels [22]. Targeted educational interventions are necessary to address specific knowledge gaps across different groups of HCPs. Although PGx content was delivered in the curricula, there is room to expand and standardize PGx content and competencies in the curricula, as recommended by the American Association of Colleges of Pharmacy (AACP) and other national organizations [33, 34].

Previous studies have reported varying levels of PGx knowledge among HCPs. Although some studies indicated fair knowledge among pharmacists and pharmacy students [35, 36], others concluded that knowledge levels are generally low among pharmacists [37]. For example, a study in Zimbabwe showed fair to good knowledge among pharmacists and undergraduate students [36]. In contrast, studies in the MENA region consistently report inadequate PGx knowledge among pharmacy and other HCPs. A study in Saudi Arabia found inadequate PGx knowledge among physicians [38], whereas another study in the United Arab Emirates (UAE) discovered a lack of adequate knowledge and awareness of PGx and its application among HCPs, including pharmacists and physicians [39]. Similarly, articles in our review reported low to moderate levels of knowledge.

The studies in our review employed various assessment scales to evaluate PGx knowledge. A systematic review of global PGx education reported that 22 studies showed a significant increase in participants' knowledge after exposure to PGx content [7]. Despite the lack of a standardized assessment scale, previous studies used similar approaches based on objective questions [40, 41, 42] or participants' perceived knowledge levels [35, 36, 43]. The choice of scoring method may have influenced the observed trends. For instance, self‐reported assessments might reflect participants' confidence in their PGx knowledge rather than their actual understanding. This highlights the importance of implementing performance‐based assessments to accurately assess the impact of educational interventions on students and HCPs' PGx competencies.

### 4.3 Key Barriers to PGx Implementation in Clinical Practice

Our review found that knowledge and education‐related barriers are the primary barriers to implementing PGx in clinical practice. This aligns with previous studies that highlighted a lack of PGx knowledge among pharmacists and physicians as a major barrier [10, 39]. A global review of barriers to PGx clinical implementation reported “education” as the most influential barrier, consistent with our findings. This barrier affects all parties involved in PGx implementation. The major sources of educational barriers include lack of PGx knowledge, lack of awareness, and inexperience in integrating PGx information into decision‐making. Additionally, there is a lack of confidence in providing services and concerns about supportive evidence to demonstrate credibility, feasibility, and clinical utility of PGx tests. These align with our findings on evidence‐based practice barriers. Barriers of availability and cost of the testing were mentioned, which is consistent with our findings on economic and infrastructure barriers [44]. Other barriers that were not explicitly reported in our studies included information technology, ethical, legal, and social issues (ELSI), regulations, and reimbursement [10]. Another scoping review on barriers to genetic testing implementation identified difficulties in interpreting and translating results of multi‐gene panels and for multi‐morbid patients [8].

### 4.4 Advancing PGx Education in the MENA Region: Recommendations and Strategies

The studies collectively emphasize the need for systematic changes in curricula, ongoing education, and collaboration to overcome barriers to PGx implementation in the MENA region. Key recommendations include the integration of PGx into existing curricula of medical and pharmacy programs [20, 21, 23, 24, 25, 26], the development of education and training programs for both students and practitioners [20, 21, 23, 25], and fostering collaboration among stakeholders, including policymakers, educators, and healthcare institutions [21, 23]. Furthermore, the importance of ongoing training, awareness campaigns, and the creation of dedicated courses is highlighted across multiple studies [20, 23, 24, 25, 26]. A study in Qatar emphasized the importance of adopting global best practices and tailoring them to local contexts to enhance PGx education and practice in the MENA region [44].

Future research should focus on initiating a region‐wide survey to map the current landscape of PGx education in the MENA region. This survey should include medical, pharmaceutical, and other health professional programs in the MENA region and analyze existing PGx curricula, identify gaps, and assess the readiness of educational institutions to adopt and integrate PGx principles. Moreover, a standardized scoring system to evaluate the proficiency of HCPs and students in PGx should be designed to measure the depth of understanding and practical application of PGx knowledge. Furthermore, in addition to perceived knowledge, the actual knowledge acquisition should be evaluated by implementing practical assessments that reflect real‐world scenarios.

## 5 Limitation

Our scoping review was limited to English‐language articles published until June 2023, with most studies published after 2020. This potentially excludes relevant studies in other languages or more recent publications. The variability in the quality of included studies and the scales used to assess knowledge could affect the reliability of our findings. Furthermore, many studies assessed knowledge levels based on participants' self‐perception, which may not accurately reflect actual knowledge. Finally, the depth of description of PGx education in the MENA region reported in the literature may not fully capture the current state of PGx delivery and education in these countries.

## 6 Conclusion

Published studies show that PGx education is delivered mainly as an integrated course in the undergraduate pharmacy and medicine programs, yet the overall knowledge level is insufficient. As PGx implementation is hindered by the knowledge and education gaps, there is an urgent need to advance curricula to align with the educational needs of the future healthcare workforce. Future research should focus on a detailed reporting of the PGx educational landscape, developing standardized assessment tools, and evaluating actual knowledge acquisition among different professions.

## Author Contributions

Conceptualization: F.M., A.I., R.A. Data curation: A.I., R.A., L.B., F.N., S.A., O.A., F.M. Methodology/formal analysis/validation: A.I., R.A., L.B., F.N., S.A., O.A., S.R., H.Z., F.M. Project administration: F.M., A.I., R.A. Writing, review, and editing: A.I., R.A., L.B., F.N., S.A., O.A., S.R., H.Z., F.M.

## Conflicts of Interest

The authors declare no conflicts of interest.

## Data Availability

The authors have nothing to report.
